# Sustainability in surgery: implications for the future

**DOI:** 10.1111/ans.17780

**Published:** 2022-08-11

**Authors:** Gareth Weijie Crouch, Payal Mukherjee, Arthur Richardson

**Affiliations:** ^1^ Department of Plastic and Reconstructive Surgery Royal North Shore Hospital St Leonards Australia; ^2^ Institute of Academic Surgery Royal Prince Alfred Hospital Sydney Camperdown Australia; ^3^ Discipline of Surgery University of Sydney Sydney Australia; ^4^ Department of Surgery Westmead Hospital Westmead Australia

Sustainability of Australian healthcare is not assured. Health costs as a fraction of GDP continue to outstrip inflation, as demands spike from an ageing population and prolonged health crises stretch budgets.^1^ Meanwhile, available resources plateau from reductions in the proportional taxpayer and private insurance base.^1^ To ensure a sustainable future, utilization of non‐capital and capital resources in surgery need careful planning to maximize beneficence, mitigate workforce burnout and prepare for crises that further burden finite resources.

## Non‐capital resources

Non‐capital resources in surgery involves ensuring the needs of the workforce to provide surgical care to the community are met. Workforce planning has failed to co‐ordinate the complete pipeline from training medical graduates to employment of surgeons in the public sector. This has created a challenge to ensure an *appropriate workforce* is present in an *appropriate geographic distribution* for equitable health care access.

### Appropriate workforce

The surgical workforce is modelled by governments to meet future demands. However, there is no systematic co‐ordination between the university sector that trains medical graduates, federal and state governments or RACS to plan public funding to Specialist Training Positions (STP) to match the number of medical graduates interested in surgical training. A lack of uniformity of points criteria for approved courses for Surgical education and training (SET) application set by different specialty training boards reduce transferability between specialties for junior trainees, prolongs entry and disincentivise diversity of prior experience in the applicants for surgical training which may not ultimately reflect the necessary scope of practice required to service the community. Consequently, a boost in medical graduate training without proportionate funding for surgical training or services has created unprecedented pressures on applicants for surgical training who take longer to enter training, face increased career uncertainty and are vulnerable to exploitation.^2^, ^3^ State and federal modelling vary, which creates a conflict between workforce demand at a local level and funding for workforce training which is done at a federal level.

### Appropriate geographic distribution

Reduced access for younger fellows in the public sector incentivises additional post‐fellowship training to qualify for highly specialized but limited metropolitan positions. Thus, fellows who complete training at an older age, with more academic qualifications, larger debt, less mobile families and reduced job opportunities in the public sector are incentivized to work in geographical regions with improved socioeconomic status, potentially increasing health care access inequity.^4^ Limited prior exposure to rural practice, predominant subspecialty metropolitan training positions and poor networks to access senior support reduce preparedness to seek rural generalist roles.^5^ In addition, without concurrent employment opportunities for partners in a small rural town, relocation is challenging. International Medical Graduates have been incentivized to fill this workforce shortage, however, while this solves the short‐term problem it disincentivises against system wide change in surgical training to promote rural preparedness for domestic workforce. The pressures on the regional surgical workforce therefore grow unsustainably, worsening burnout and attrition.^6^

Investment in training represents a large non‐capital cost. However, reducing attrition and burnout ensures return on investment with estimated savings in the US of USD $6.3 billion.^7^ Surgeons lost to the private system similarly represent a loss of value, raising the question whether taxpayer investment in workforce training is delivering equitable service provision and value to the community.^7^

## Capital resources

Paralleling untenable pressures on human resources is a critical unsurpassed demand on limited capital resources. As costs of new interventions rise and surgical budgets strain, health systems can respond by (i) reducing unnecessary cost by implementing value‐based healthcare models,^8^ (ii) ensuring stringent heath technology assessment of new interventions and (iii) mitigating conflicts of interests and industry pressures in adoption of new technology. In NSW public hospitals alone, up to 8855 procedures of questionable benefit are performed yearly, costing approximately AUD $99.3 million.^9^ Opaque supply systems with poor stock visibility contribute to oversupply representing waste, or undersupply delaying timely care, as demonstrated by PPE shortages early in the COVID pandemic. Consumable costs vary by a factor of 10 between hospitals due to inconsistent contracts and reduced purchasing power due to the variety of surgeon‐requested consumables.^10^ Industry funded training for use of surgical interventions can create conflicts of interests which may drive key stakeholders in surgery to support funding models for health technology without evidence. Volume based funding incentivises matching metrics against key performance indicators (KPIs) that promote shorter term, less sustainable solutions. Value‐based funding of health care may assist in mitigating these pressures.

## Resource preservation by mitigating crisis

Crisis management increases demand on capital and non‐capital resources and exacerbates health inequity. As seen during COVID‐19, crises necessitate prioritization of acute health care, which has a disproportionate impact on non‐urgent surgery. If the system is overwhelmed with numerous crises prolonging the impact of the shock to routine care, overcoming subsequent backlogs places unsustainable pressure on the workforce which impacts rural areas and areas with poor socioeconomic status due to limited workforce availability and high demand on public health services. Public‐private partnerships globally have been utilized as a short‐term strategy to overcome backlogs but they divert money away from development of local infrastructure and workforce expertise needed to deliver longer‐term sustainable public care.^11^ Moreover, patients forego longer‐term follow‐up if their surgeon is an interstate locum, and waitlists of local rural surgeons may potentially become dominated by complex or revision operations.

Environmental resources, not typically considered as financial costs, are hidden areas of unsustainable cost. Climate emergencies such as the bushfires of 2019 have demonstrated the magnitude of this on workforce and health care burden, estimated in the order of hundreds of billions of dollars.^12^ Steps must be taken to mitigate future crises but also address contribution from surgery to the problem. Operating theatres contribute 3–6 times the greenhouse gas compared to elsewhere in the hospital and 20–30% of the 42 000 tons of solid waste produce by Victorian public hospitals in 2019.^13^, ^14^ Reliance on single use plastics also has less‐obvious value implications, including vulnerability to supply variation, illustrated by 2020's global shortage of sterilization wraps that threatened Australian surgical services.

## Conclusion

Increasing concern about the sustainability of Australian surgical care necessitates measures to reduce waste as an economic and ethical priority. COVID‐19 has provided a unique opportunity to press the reset button, examine areas of weakness and implement change (Table 1). It is clear there are many opportunities to finding value in healthcare – the question is how. Solutions will be multifaceted, and in the words of General Boyd, should include ‘People. Systems. Equipment! In that order!’

**TABLE 1:** Measures to promote sustainability in our health system need to support the people within it, systems that run it and equipment that resource it

	People	System	Equipment
Reduce low‐value care	Define low value care (e.g. “Do not perform” lists, decision support tools) to assist clinicians in choosing treatment Provide performance feedback to clinicians	Publish hospitals' rates of low‐value care Consolidate elective surgery into fewer, easily governable centres Substitute inpatient care with lower out of hospital care Encourage value‐based renumeration	Develop multicentre procedure databases, with AI searches to detect low value healthcare Use wearable technology to facilitate outpatient care and artificial intelligence assisted patient flow systems to improve flow Deploy blockchain communication systems between care teams to prevent unnecessary tests
Increase translational speed of high value care	Appoint clinicians based on contribution to translational research	Develop rapid‐access pathways which facilitate access to new technologies Encourage training pathways for new technologies Develop consent tools to facilitate ethical trial recruitment	Use multi‐institutional longitudinal databases to pool evaluation data of new technologies more rapidly
Reduce complexity in the supply chain	Encourage individual surgeons to reconsider the consumables they choose	Standardize consumable use Develop centralized procurement and supply chain systems	Develop transparent information control systems that track real‐time equipment use
Encourage equitable workforce distribution	Select champions of rural health Select rural trainees and medical students Support younger fellows and families setting up in rural sectors	Ensure training schemes encourage rural participation and train for the challenges of rural care Establish remote career coordination programs Ensure matched numbers of training positions to graduates Ensure hospital budgets for recruitment align with state/federal workforce planning models	Investigate virtual reality solutions to ensure adequate supervision or exposure in rural environments Deploy adequate teleconference services so rural surgeons retain access to metropolitan educational opportunities
Reduce natural resource use	Appoint leaders to navigate environmental change Encourage individual reduction of single use equipment, and inhaled anaesthesia	Repackage surgical trays to minimize redundancy Develop College supported sustainable development units across specialties Introduce green targets across the hospital Assess environmental impact during procurement processes	Adopt energy‐saving and green technologies, for example, low consumption light bulbs, solar panels Deploy e‐health, live data sharing and teleconferencing software for better communication across levels of care and prevent duplication of care/investigations
Reduce human turnovers	Measure and educate on signs of burnout Encourage individuals to take regular leave Target perpetrators of bullying and harassment	Systematically screen for burnout Build in redundancy that allows surgeons to take leave Develop flexible training schemes	Use new technology e.g. speech recognition to reduce administrative load that contributes to burnout

## Author contributions

**Gareth Weijie Crouch:** Conceptualization; methodology; writing – original draft; writing – review and editing. **Payal Mukherjee:** Conceptualization; methodology; writing – original draft; writing – review and editing. **Arthur Richardson:** Conceptualization; supervision; writing – review and editing.
